# Association of small intestinal bacterial overgrowth with portal hypertension in HCV-related cirrhosis: a cross-sectional study

**DOI:** 10.1007/s10238-025-01871-0

**Published:** 2026-03-16

**Authors:** Tary Salman, Gasser El-Azab, Fatma Khalil, Reham Elkazaz, Abdelaleem Helal

**Affiliations:** 1 Hepatology and Gastroenterology Department, National Liver Institute, Menoufia University, Shebeen El-Kom, 32511 Menoufia Egypt; 2 Clinical Microbiology and Immunology Department, National Liver Institute, Menoufia University, Shebeen El-Kom, Egypt

**Keywords:** Small intestinal bacterial overgrowth, Cirrhosis, Portal hypertension, HCV, Hepatic encephalopathy, FIB-4, MELD

## Abstract

Small intestinal bacterial overgrowth (SIBO) is a frequent and clinically significant complication in patients with liver cirrhosis. However, its association with portal hypertension (PH) in HCV-related cirrhosis remains underexplored. To determine the prevalence, predictors, and clinical associations of SIBO in patients with HCV-related cirrhosis, with particular emphasis on its relationship to portal hypertension. In this cross-sectional study, we evaluated 90 patients with HCV-related cirrhosis and 30 control subjects without liver disease. SIBO was diagnosed using quantitative duodenal aspirate cultures. Clinical, laboratory, and endoscopic data were collected. Multivariate logistic regression was performed to identify independent predictors of SIBO. SIBO was detected in 63% of cirrhotic patients with portal hypertension, 41.7% of those without portal hypertension, and 6.7% of controls (*p* < 0.001). Detectable HCV RNA was significantly associated with higher SIBO prevalence and increased bacterial colony counts (*p* < 0.001). The most frequently isolated organisms were Enterococcus faecalis and Streptococci. Multivariate analysis identified age (OR = 1.09, *p* = 0.002), FIB-4 (OR = 1.61, *p* = 0.001), MELD score (OR = 1.15, *p* = 0.005), and portal hypertension (OR = 2.89, *p* = 0.048) as independent predictors of SIBO. SIBO is highly prevalent in HCV-related cirrhosis, especially in patients with portal hypertension and ongoing HCV replication. Age, FIB-4, MELD, and portal hypertension are independent predictors of SIBO. Screening for and managing SIBO may be particularly important in patients with advanced liver disease, especially those with portal hypertension.

## Introduction

Small Intestinal Bacterial Overgrowth (SIBO) is increasingly recognized as a significant contributor to the pathophysiology of chronic liver disease (CLD), particularly in patients with cirrhosis and portal hypertension. SIBO refers to an abnormal increase in the microbial population within the small intestine, which disrupts gut homeostasis and contributes to systemic complications through bacterial translocation and inflammatory pathways [1]. The prevalence of SIBO in patients with CLD is notably high, with a pooled analysis estimating an eightfold increase compared to healthy controls [2].

In cirrhosis, progressive liver dysfunction leads to alterations in the gut-liver axis, including intestinal dysbiosis, increased gut permeability, and impaired immune defenses. These changes facilitate bacterial translocation from the gut lumen into systemic circulation, exacerbating complications such as portal hypertension, hepatic encephalopathy (HE), and spontaneous bacterial peritonitis (SBP) [3]. A systematic review demonstrated that SIBO prevalence was significantly higher in patients with decompensated cirrhosis compared to those with compensated cirrhosis (odds ratio (OR) = 2.6; 95% CI 1.5–4.5), underscoring its association with disease progression.

Portal hypertension itself has been implicated as a key driver of gut dysbiosis in cirrhosis. A study analyzing gut microbiota composition revealed significant differences between patients with portal hypertension and healthy controls, highlighting an increase in pathogenic bacteria such as Streptococcus and Enterococcus and a reduction in beneficial commensals like Faecalibacterium and Roseburia [4]. In another study, SIBO was found to be more prevalent in patients with portal hypertension (OR = 2.1; 95% CI 1.4–3.1), suggesting that portal hypertension may exacerbate intestinal dysbiosis or vice versa. Among complications of portal hypertension, SBP had the highest prevalence of SIBO at 57.7%, followed by HE at 41.0% and variceal bleeding at 39.5% [2].

Despite these insights, the interplay between SIBO and portal hypertension remains incompletely understood. While portal hypertension appears to drive gut dysbiosis through mechanisms such as intestinal congestion and impaired motility [4], it is also plausible that SIBO exacerbates portal hypertension by contributing to systemic inflammation and endothelial dysfunction [5]. This bidirectional relationship warrants further investigation using robust diagnostic methods such as duodenal aspirate cultures, which provide definitive evidence of bacterial overgrowth compared to less specific tests like breath analysis [6].

This study aims to explore the association between SIBO and portal hypertension in hepatitis C virus (HCV)-related cirrhosis using duodenal aspirate cultures for SIBO diagnosis.

## Patients and methods

### Study type

This was a cross-sectional observational study. Participants with HCV-related cirrhosis were divided into groups based on their portal hypertension status, in addition to a control group undergoing endoscopy for non-liver disease indications.

### Study population

The study population included adults (≥ 18 years old) and comprised three groups.

#### Group 1: non-liver disease controls

This group included adults undergoing upper GI endoscopy for reasons other than known or suspected liver disease or portal hypertension.

#### Group 2: HCV-related cirrhosis without portal hypertension

This group included patients with a confirmed diagnosis of HCV-related cirrhosis who exhibited no clinical, laboratory, or imaging evidence of portal hypertension.

#### Group 3: HCV-related cirrhosis with portal hypertension

HCV-related liver cirrhosis was diagnosed based on histological evidence from liver biopsy (if available), clinical signs of cirrhosis, and/or FibroScan with a liver stiffness measurement (LSM) > 12.5 kPa [7, 8]. Patients were also positive for HCV antibody and had detectable HCV RNA (or had a history of successful treatment for HCV).

Portal hypertension was defined by at least one of the following criteria: esophageal or gastric varices identified on endoscopy, history of variceal bleeding, ascites (current or past history), LSM ≥ 20 kPa, splenomegaly (spleen size > 13 cm by ultrasound), or platelet count < 150,000/μL [9, 10].

All groups shared the following exclusion criteria: history of other chronic liver diseases (e.g., autoimmune hepatitis, primary biliary cholangitis, hemochromatosis), co-infection with hepatitis B virus (HBV) or human immunodeficiency virus (HIV), history of gastrointestinal surgery (e.g., gastric resection, bariatric surgery, small bowel resection), active malignancy, inflammatory bowel disease, use of antibiotics, probiotics, prebiotics, or intestinal motility agents within 3 months before enrollment, recent gastrointestinal bleeding, use of proton pump inhibitors (PPIs) or H2-receptor antagonists within 1 month before enrollment, pregnancy or breastfeeding, diabetes mellitus, and significant recent alcohol intake (defined as > 20 g/day for females and > 30 g/day for males within the past 6 months) or active drug abuse.

### Recruitment and enrollment

Participants were recruited from the endoscopy unit, Gastroenterology and Hepatology Department, National Liver Institute, Menoufia University, Egypt. Potential participants were screened for eligibility based on inclusion/exclusion criteria, and eligible patients were invited to participate in the study and provided written informed consent.

### Demographic and clinical data

Data collected included age, sex, medical history, medications, alcohol and tobacco use, and history of liver disease complications (e.g., ascites, variceal bleeding, hepatic encephalopathy) for cirrhosis groups. Information on prior HCV antiviral therapy and sustained virological response (SVR) status was also obtained. The physical examination included the assessment of ascites, splenomegaly, and other signs of liver disease for the cirrhosis groups. Body mass index (BMI) was calculated as weight (in kilograms) divided by height (in meters) squared. In patients with ascites, BMI was adjusted using estimated dry weight, obtained by subtracting 5%, 10%, or 15% of body weight for mild, moderate, or severe ascites, respectively, with an additional 5% deducted if bilateral pedal edema was present [11, 12].

### Laboratory data

The laboratory data included Complete blood count (CBC) with differential, FBS and Hba1c, Comprehensive metabolic panel (CMP), including liver function tests (ALT, AST, alkaline phosphatase, bilirubin, albumin), INR, alpha-fetoprotein (AFP), Renal function tests (creatinine, BUN) and Viral markers (HCV Ab/PCR, HBsAg), C-reactive protein (CRP).

### Abdominal ultrasound and liver stiffness measurement

All patients underwent abdominal ultrasonography using a GE LOGIQ 9 machine. Liver stiffness measurement was performed using FibroScan with the FibroScan Medium (M +) probe and 5.02 Touch operating software (Echosens, France). The procedure followed established guidelines, ensuring at least 10 valid measurements per patient. Liver stiffness values were expressed in kilopascals (kPa), and the controlled attenuation parameter (CAP) values were recorded to assess hepatic steatosis.

### Assessment of portal hypertension and severity of liver disease

Portal hypertension was assessed clinically based on ascites and laboratory parameters, including platelet count. Imaging assessments included measurement of splenic size, Doppler ultrasound to evaluate portal vein diameter and assess for portosystemic shunts [13], and FibroScan to measure liver stiffness [8]. Esophagogastroduodenoscopy (EGD) was performed to detect esophageal and gastric varices [14]. Additionally, the Child–Pugh and Model for End-Stage Liver Disease (MELD) scores were computed to assess liver disease severity [15, 16]. Non-invasive fibrosis scores, including the APRI (AST to Platelet Ratio Index) and FIB-4 scores, were also calculated [17].

### Upper endoscopy and duodenal aspiration

EGD was performed to assess for esophageal and gastric varices (for cirrhosis groups), with varices graded according to Paquet classification [18]. Endoscopic procedures were conducted by experienced operators who were not involved in outcome assessment or data analysis. During EGD, a duodenal aspirate was collected using a sterile technique to minimize contamination. Before the EGD procedure, patients were required to perform their usual oral hygiene, including cleaning the mouth, tongue, and teeth, rinsing their mouth, and gargling their throat with a hexedine solution. Esophagogastroduodenoscopy (PENTAX EG29-i10) was performed with the patient in the left lateral position under light sedation with midazolam. A gastroscope fitted with a double-lumen catheter was used to collect luminal fluid samples from the duodenum. The catheter assembly consisted of two tubes, with the inner tube being 3 cm longer than the outer tube, whose mouth was sealed by an obturator. The endoscopist advanced the scope to the second portion of the duodenum and introduced the catheter through the biopsy channel. The inner catheter was then used to aspirate 1–2 mL of duodenal fluid. The aspirated fluid was collected through the inner tube using a sterile syringe and was transported to the microbiology laboratory for culture and sensitivity testing.

### Microbiology analysis

Samples were transported at room temperature and delivered to the microbiology laboratory within three hours of collection. Upon arrival, they were immediately processed for quantitative culture, including aerobic and anaerobic bacterial counts, yeast cultures, and antibiotic sensitivity testing. For anaerobic cultures, samples were plated on kanamycin-vancomycin (KV) blood agar and incubated at 37 °C in a GasPak 100 anaerobic system. Colonies were counted after 48 h. Aerobic cultures were plated on Levine eosin-methylene blue agar (Becton Dickinson) and incubated at 37 °C for 24 h before colony counting. Total bacterial colony counts from all culture conditions were summed and normalized to the volume of undiluted luminal fluid to calculate total colony-forming units per milliliter (CFU/mL) [19, 20]. The microbiologist analyzing duodenal aspirate cultures was blinded to patients' group assignments to minimize observer bias.

SIBO was defined as a total bacterial count of ≥ 10^3^ CFU/mL in the duodenal aspirate [21, 22]. Identified bacterial species were recorded.

### Outcome measures

The primary outcome of this study was to compare the prevalence of SIBO in patients with HCV-related cirrhosis, stratified by the presence or absence of portal hypertension.

Secondary outcomes included: identifying specific bacterial species in duodenal aspirates associated with portal hypertension, examining the association between the presence and bacterial load of SIBO (CFU/mL) and history of HE, and exploring potential risk factors for SIBO in patients with HCV-related cirrhosis.

### Statistical analysis

Data were analyzed using IBM SPSS Statistics, version 25.0 (IBM Corp., 2017). Normality of data distribution was assessed using the Shapiro–Wilk and Kolmogorov–Smirnov tests. A two-tailed *p* value < 0.05 was considered statistically significant.

Continuous variables were summarized as means ± standard deviations for normally distributed data, or medians with ranges or interquartile ranges (IQRs) for non-normally distributed data. Categorical variables were presented as frequencies and percentages. The chi-square test was used for group comparisons, while Fisher’s exact test was applied to tables with small expected counts. The Monte Carlo simulation method was applied to contingency tables with sparse data to approximate exact p-values. Continuous variables were compared across groups using one-way ANOVA for normally distributed data, followed by Tukey’s post hoc test for pairwise comparisons. For non-normally distributed data, the Kruskal–Wallis test was used, with Dunn’s post hoc test for pairwise comparisons. Comparisons between patients with and without SIBO were conducted using the independent-samples t-test or the Mann–Whitney U test for continuous variables, and the chi-square test or Fisher’s exact test for categorical variables.

To identify independent predictors of SIBO among cirrhotic patients, binary logistic regression analysis was performed using the backward stepwise (conditional) method. Variables with statistically significant associations in univariate analysis were entered into the multivariate model.

### Sample size calculation

A sample size calculation was performed to determine the required number of participants in each group based on the expected prevalence of SIBO and the desired statistical power. The study aimed for 80% power (β = 0.20) with a 5% significance level (α = 0.05). A minimum of 30 participants per group was required to ensure sufficient statistical power.

### Ethical considerations

The study protocol was reviewed and approved by the Institutional Review Board (IRB) at the National Liver Institute, Menoufia University, Egypt (approval number: 00587/2024). Informed consent was obtained from all participants before enrollment. Patient confidentiality was maintained throughout the study. Participants were informed of their right to withdraw from the study at any time without penalty. Any serious adverse events were reported to the IRB and the appropriate regulatory authorities.

## Results

### Demographic and clinical characteristics

Baseline demographic and clinical characteristics of the study groups are presented in Table 1. There were no statistically significant differences in age, sex distribution, or BMI among the groups. However, liver stiffness differed significantly (*p* < 0.001). Patients with cirrhosis and portal hypertension (Group 3) exhibited the highest liver stiffness (24.0 ± 3.12 kPa), reflecting more advanced liver disease. Clinical complications, including ascites, varices, and a history of hepatic encephalopathy, were significantly more frequent in Group 3 compared to Group 2 (*p* < 0.01).

**Table 1:** Demographic and clinical characteristics of the studied groups

Variable	Non-Liver Disease Controls (n = 30) Group 1	Cirrhosis without PH (n = 36) Group 2	Cirrhosis with PH (n = 54) Group 3	Test of sig	*p* value
Age (years), mean ± SD	48.57 ± 13.032	50.17 ± 12.246	54.65 ± 10.974	F = 2.99	P_0_ = 0.055
Male sex, n (%)	15 (50.0%)	14 (38.9%)	24 (44.4%)	χ^2^ = 0.822	P_0_ = 0.663
BMI*, mean ± SD	27.6 ± 5.04	26.33 ± 4.51	25.76 ± 4.72	F = 1.453	P_0_ = 0.238
Ascites, n (%)	N/A	0 (0.0%)	18 (33.3%)	χ^2^ = 15	P_0_ < 0.001
Varices, n (%)	N/A	0 (0.0%)	28 (51.8%)	χ^2^ = 22.474	P_0_ < 0.001
H/O HE, n (%)	N/A	6 (16.7%)	24 (44.4%)	χ^2^ = 7.5	P_0_ = 0.006
Liver stiffness (kPa), mean ± SD	4.75 ± 1.28	16.71 ± 1.56	24 ± 3.12	H = 98.012	P_0_ < 0.001 p_1_ < 0.001 p_2_ < 0.001 p_3_ < 0.001

F: One-way ANOVA test, H: Kruskal–Wallis test, χ^2^: Chi-square test, SD: Standard deviation

BMI: Body mass index, *: BMI was calculated using estimated dry weight in ascitic patients. H/O HE: history of hepatic encephalopathy

P_0_: *p* value for comparing the studied groups

p_1_: *p* value for comparing Group 1 and Group 2

p_2_: *p* value for comparing Group 1 and Group 3

p_3_: *p* value for comparing Group 2 and Group 3

### Laboratory results

Laboratory parameters demonstrated significant differences across the three study groups (Table 2). Patients with cirrhosis and portal hypertension (Group 3) showed the most severe derangements: marked thrombocytopenia (114.13 ± 28.32 × 10⁹/L vs. 165.3 ± 28.58 in Group 2 and 238.7 ± 57.34 in controls; *p* < 0.001), hypoalbuminemia (3.05 ± 0.37 g/dL vs. 4.04 ± 0.60 and 4.49 ± 0.48; *p* < 0.001), and elevated total bilirubin (2.36 ± 1.04 mg/dL vs. 1.16 ± 0.40 and 0.90 ± 0.20; *p* < 0.001). Group 3 also demonstrated higher AST (64.65 ± 27.86 U/L), INR (1.58 ± 0.33), and creatinine (1.37 ± 0.63 mg/dL) versus other groups (*p* ≤ 0.001). Hemoglobin reduction was significant in both cirrhosis groups versus controls (*p* < 0.001), while detectable HCV RNA prevalence did not differ between Group 2 (72.2%) and Group 3 (74.1%, *p* = 0.845).

**Table 2:** Laboratory results of the studied groups

Variable	Non-Liver Disease Controls (n = 30) Group 1	Cirrhosis without PH (n = 36) Group 2	Cirrhosis with PH (n = 54) Group 3	Test of sig	*p* value
*HB (g/dl)*					
Min.–Max	9.10–15.0	7.30–13.20	6.80–14.40	F = 13.420	P0 > 0.001 p1 > 0.001 p2 > 0.001 p3 = 0.920
Mean ± SD	12.21 ± 1.34	10.28 ± 1.60	10.42 ± 1.92		
Median (IQR)	12.30(11.0–13.20)	10.0 (9.0–11.80)	10.60(8.90–11.70)		
WBC (× 10^3^/μl)					
Min.–Max	3.60–7.90	2.50–12.0	2.50–7.30	F = 11.396	P0 > 0.001 p1 = 0.401 p2 = 0.011 p3 > 0.001
Mean ± SD	6.03 ± 1.17	6.48 ± 1.82	5.11 ± 1.12		
Median (IQR)	6.15 (5.20–6.80)	6.20 (5.30–7.55)	5.10 (4.40–6.0)		
*Platelet count (*× *10*^*9*^*/L)*					
Min.–Max	165.0–350.0	113.0–221.0	50.0–198.0	F = 105.549	P0 > 0.001 p1 > 0.001 p2 > 0.001 p3 > 0.001
Mean ± SD	238.7 ± 57.34	165.3 ± 28.58	114.1 ± 28.32		
Median (IQR)	219.5(195.0–290.0)	165.5(145.0–185.5)	118.5(98.0–132.0)		
*Total Bilirubin (mg/dL)*					
Min.–Max	0.50–1.20	0.60–2.40	0.60–5.80	H = 64.441	P0 > 0.001 p1 = 0.037 p2 > 0.001 p3 > 0.001
Mean ± SD	0.90 ± 0.20	1.16 ± 0.40	2.36 ± 1.04		
Median (IQR)	0.90 (0.70–1.10)	1.10 (0.90–1.20)	2.30 (1.50–3.0)		
*Direct bilirubin (mg/dL)*					
Min.–Max	0.30–0.90	0.16–1.80	0.10–4.70	H = 46.522	P0 > 0.001 p1 = 0.090 p2 > 0.001 p3 > 0.001
Mean ± SD	0.51 ± 0.15	0.66 ± 0.33	1.52 ± 0.87		
Median (IQR)	0.46 (0.40–0.59)	0.60 (0.45–0.75)	1.50 (0.90–1.90)		
*Albumin (gm/dL)*					
Min.–Max	3.90–5.50	3.10–5.30	2.10–3.70	F = 100.816	P0 > 0.001 p1 = 0.001 p2 > 0.001 p3 > 0.001
Mean ± SD	4.49 ± 0.48	4.04 ± 0.60	3.05 ± 0.37		
Median (IQR)	4.45 (4.10–4.80)	3.95 (3.60–4.55)	3.10 (2.90–3.30)		
*AST (U/L)*					
Min.–Max	11.0–32.0	15.0–103.0	17.0–146.0	H = 67.330	P0 > 0.001 p1 = 0.007 p2 > 0.001 p3 > 0.001
Mean ± SD	20.83 ± 5.07	31.69 ± 19.04	64.65 ± 27.86		
Median (IQR)	21.0(17.0–24.0)	27.0(22.0–30.50)	61.50(47.0–78.0)		
ALT (U/L)					
Min.–Max	11.0–30.0	11.0–170.0	11.0–126.0	H = 30.648	P0 > 0.001 p1 = 0.020 p2 > 0.001 p3 = 0.002
Mean ± SD	20.77 ± 5.08	30.86 ± 26.04	47.69 ± 25.88		
Median (IQR)	21.0(18.0–24.0)	26.0(21.0–30.0)	45.0(26.0–67.0)		
*INR*					
Min.–Max	0.80–1.20	0.80–2.10	1.0–2.50	H = 62.971	P0 > 0.001 p1 = 0.075 p2 > 0.001 p3 > 0.001
Mean ± SD	0.99 ± 0.14	1.14 ± 0.31	1.58 ± 0.33		
Median (IQR)	1.0 (0.90–1.10)	1.10(0.90–1.20)	1.50(1.37–1.72)		
*Creatinine (mg/dL)*					
Min.–Max	0.60–1.20	0.70–1.30	0.60–3.20	H = 14.022	P0 = 0.001 p1 = 0.720 p2 = 0.001 p3 = 0.003
Mean ± SD	0.96 ± 0.19	0.99 ± 0.18	1.37 ± 0.63		
Median (IQR)	1.0 (0.80–1.10)	1.0 (0.85–1.10)	1.20(0.90–1.60)		
Detectable HCV RNA by PCR, n (%)	N/A	26 (72.2%)	40 (74.1%)	χ^2^ = 0.038	P_0_ = 0.845

F: One-way ANOVA test, H: Kruskal–Wallis test, χ^2^: Chi-square test, SD: Standard deviation

H/O HE: history of hepatic encephalopathy

P_0_: *p* value for comparing the studied groups

p_1_: *p* value for comparing Group 1 and Group 2

p_2_: *p* value for comparing Group 1 and Group 3

p_3_: *p* value for comparing Group 2 and Group 3

### Prevalence of small intestinal bacterial overgrowth (SIBO)

**Fig. 1:**
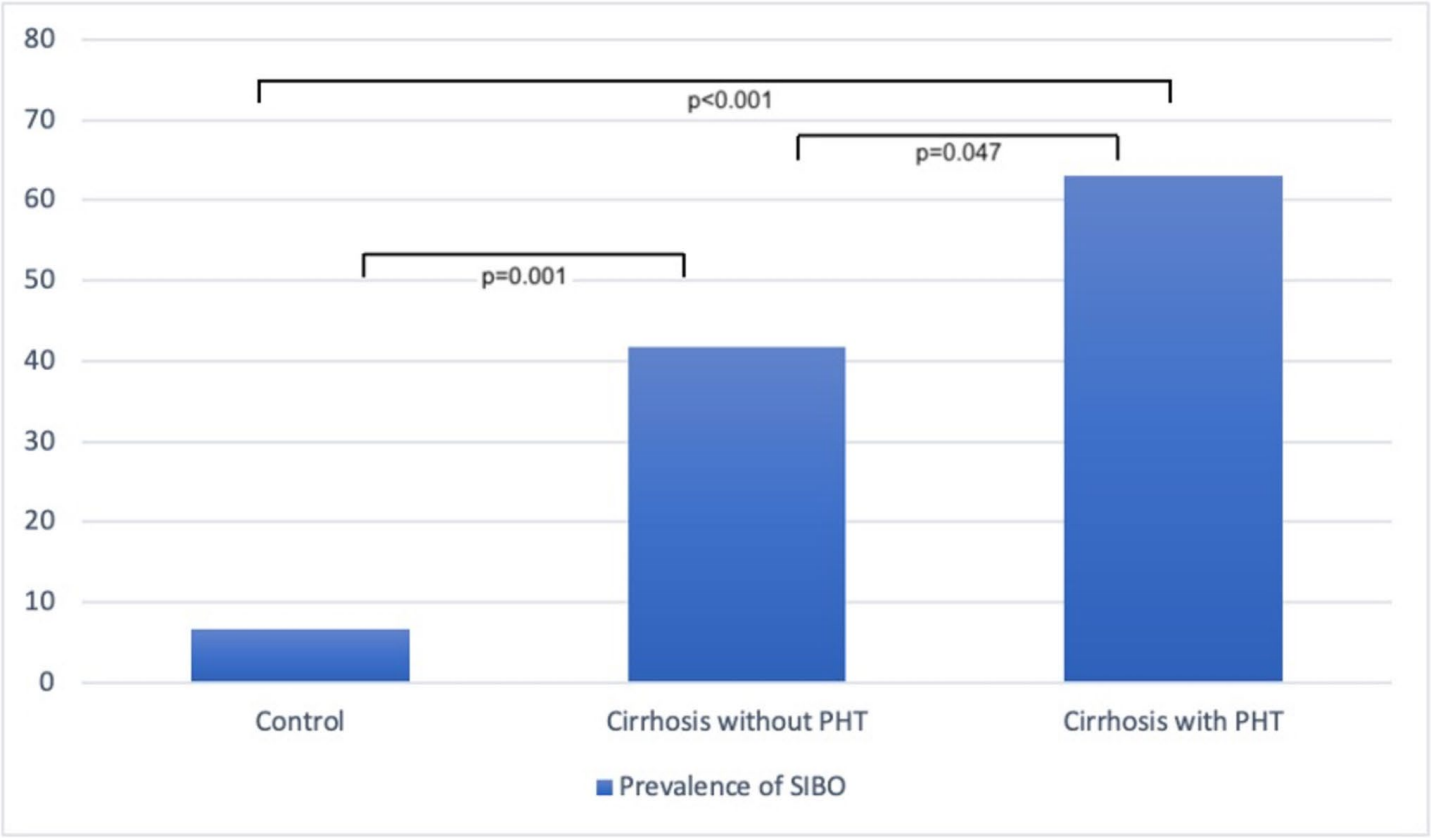
Comparison of small intestinal bacterial overgrowth among the different studied groups

As shown in Table 3 and Fig. 1, the prevalence of SIBO increased significantly across the study groups: 6.7% in Group 1, 41.7% in Group 2, and 63% in Group 3 (χ^2^ = 25.026, *p* < 0.001). Post-hoc analysis confirmed significant differences between Group 1 and both cirrhosis groups (*p* = 0.001 and *p* < 0.001, respectively), as well as between Groups 2 and 3 (*p* = 0.047).

**Table 3:** Comparison between the studied groups regarding small intestinal bacterial overgrowth (SIBO) and small intestinal microbiota colony counts

	Non-Liver Disease Controls (n = 30) Group 1	Cirrhosis without PH (n = 36) Group 2	Cirrhosis with PH (n = 54) Group 3	Statistical test	
*SIBO*				χ^2^	*p*
No, n (%)	28 (93.3)	21 (58.3)	20 (37)	25.026	P_0_ < 0.001 p_1_ = 0.001 p_2_ < 0.001 p_3_ = 0.047
Yes, n (%)	2 (6.7)	15 (41.7)	34 (63)		
*Colony count (*× *10*^*3*^* CFU/mL)*				H	*p*
Min.–Max	0.2–1.2	0.3–2.3	0.4–4	38.84	P0 < 0.001 p1 = 0.001 p2 < 0.001 p3 = 0.009
Mean ± SD	0.62 ± 0.23	1.15 ± 0.59	1.82 ± 1.08		
Median (IQR)	0.6 (0.5–0.8)	0.9 (0.8–1.75)	1.7 (0.9–2.5)		

IQR: Interquartile range, SD: Standard deviation, χ^2^: Chi-square test, H: Kruskal–Wallis test

P_0_: *p* value for comparing the studied groups

p_1_: *p* value for comparing Group 1 and Group 2

p_2_: *p* value for comparing Group 1 and Group 3

p_3_: *p* value for comparing Group 2 and Group 3

### Bacterial species identified in duodenal aspirates

**Fig. 2:**
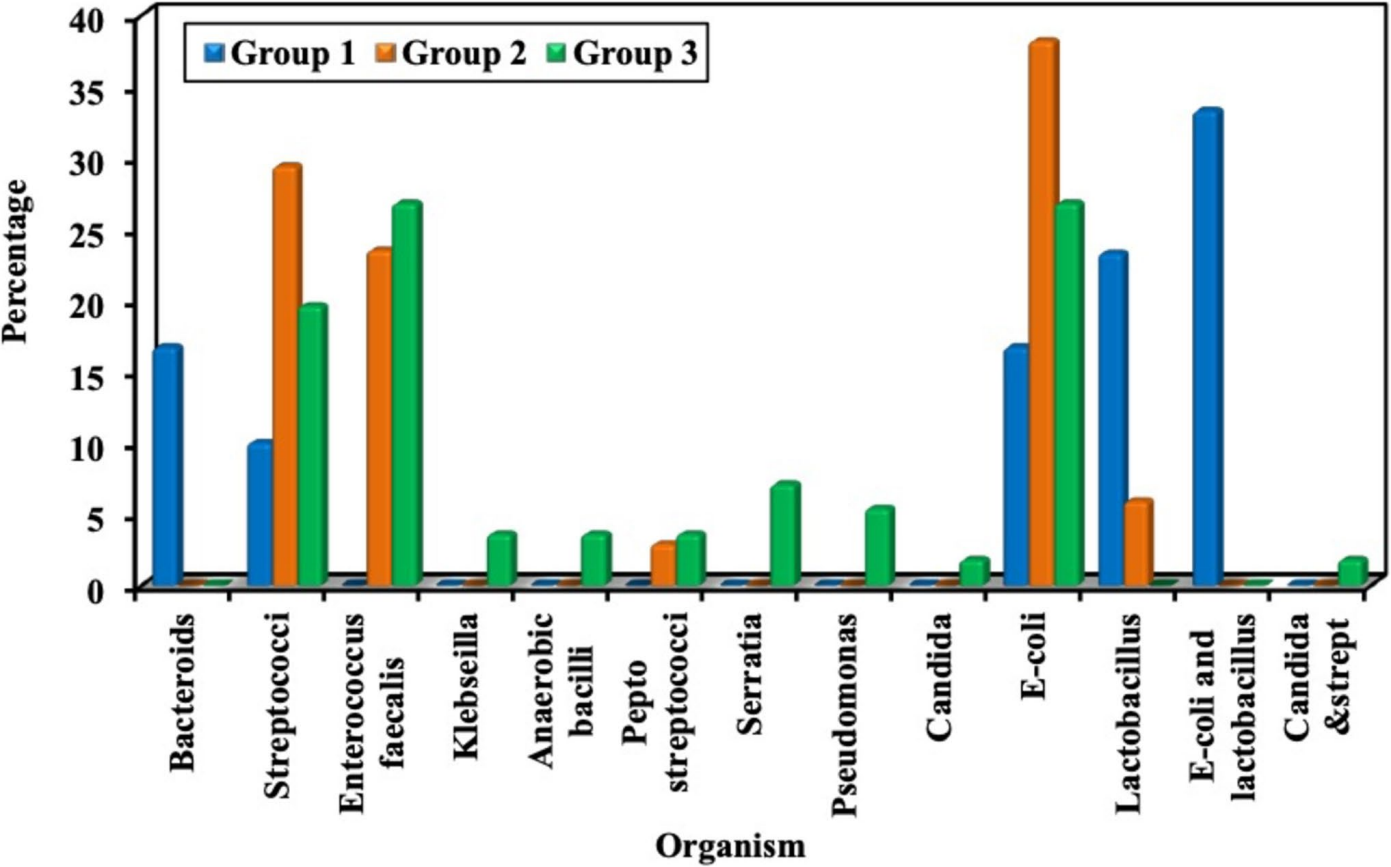
Bacterial species identified in duodenal aspirates from the studied groups

Table 4 and Fig. 2 summarize the microbial species identified in duodenal aspirates. Bacteroides and co-isolation of Escherichia coli and Lactobacillus were observed exclusively in Group 1. Lactobacilli were significantly more prevalent in Group 1 (*p* < 0.001). Enterococcus faecalis was significantly more common in Groups 2 and 3 (*p* = 0.009), while Streptococci predominated in Group 2 (*p* = 0.013). Other organisms, including Klebsiella, Pseudomonas, Serratia, and Candida, were detected only in Group 3, though these differences did not reach statistical significance.

**Table 4:** Comparison between the studied groups regarding bacterial species identified in duodenal aspirates

Organism	Groups							
	Group 1 (n = 30)		Group 2 (n = 36)		Group 3 (n = 54)		χ^2^	*p*
	No	%	No	%	No	%		
*Bacteroides*	5	16.7	0	0.0	0	0.0	10.976	^MC^p = 0.001
*Streptococci*	3	10.0	13	36.1	8	14.8	8.623	0.013
*Enterococcus faecalis*	0	0.0	9	25.0	14	25.9	9.496	0.009
*Klebsiella*	0	0.0	0	0.0	2	3.7	1.575	^MC^p = 0.492
*Anaerobic bacilli*	0	0.0	0	0.0	2	3.7	1.575	^MC^p = 0.492
*Pepto streptococci*	0	0.0	0	0.0	3	5.6	2.420	^MC^p = 0.335
*Serratia*	0	0.0	0	0.0	4	7.4	3.522	^MC^p = 0.154
*Pseudomonas*	0	0.0	0	0.0	3	5.6	2.420	^MC^p = 0.335
*Candida*	0	0.0	0	0.0	1	1.9	1.327	^MC^p = 1.000
*E-coli*	5	16.7	12	33.3	16	29.6	2.503	0.286
*Lactobacillus*	7	23.3	2	5.6	0	0.0	13.487	^MC^p < 0.001
*E-coli and lactobacillus*	10	33.3	0	0.0	0	0.0	25.616	^MC^p < 0.001
*Candida and streptococci*	0	0.0	0	0.0	1	1.9	1.327	^MC^p = 1.000

χ^2^: Chi square test, MC: Monte Carlo test

### Bacterial colony count

**Fig. 3:**
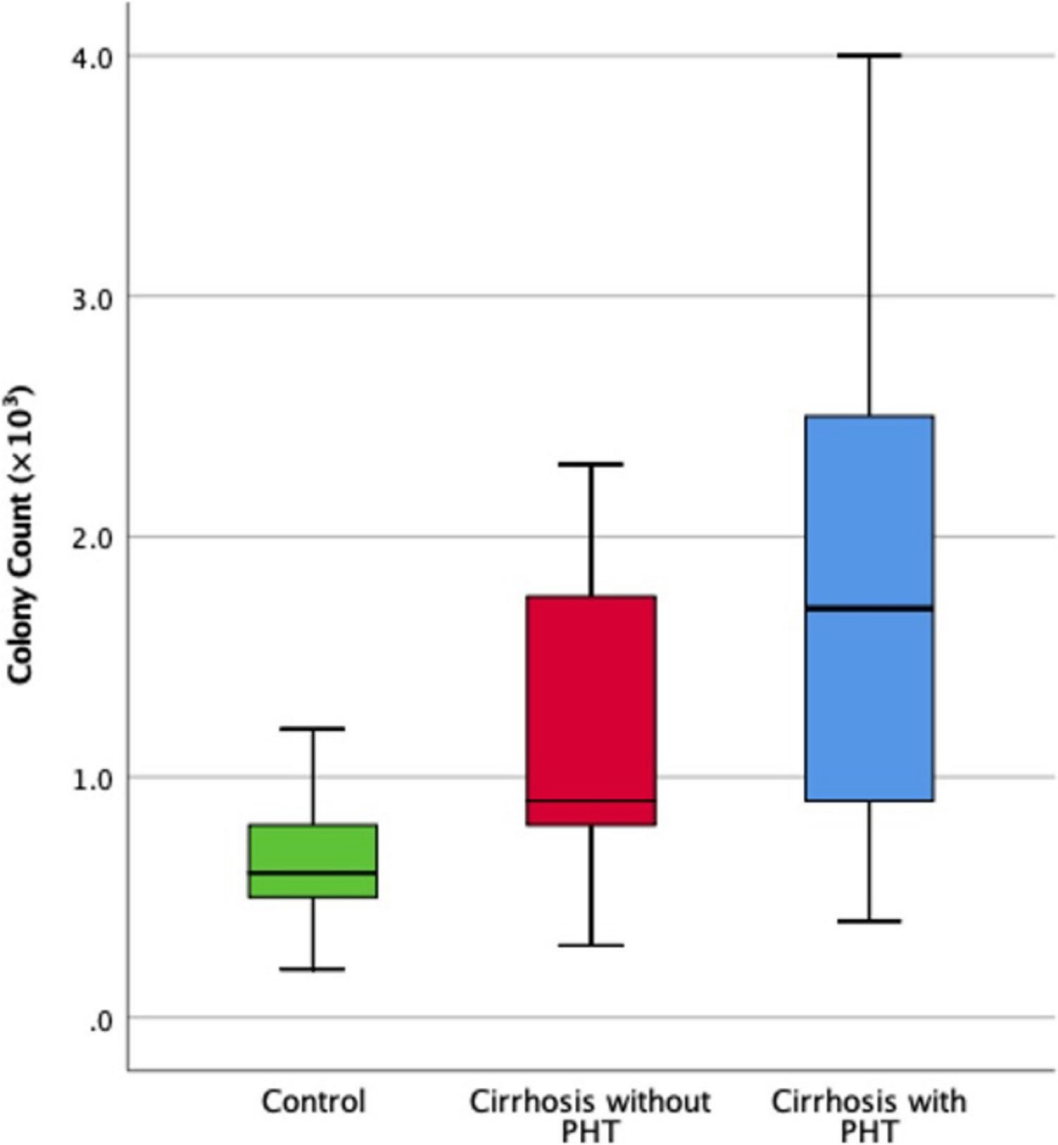
Comparison between the different studied groups according to the colony count of small intestinal microbiota

As illustrated in Table 3 and Fig. 3, there were significant differences in bacterial colony counts among the groups (*p* < 0.001). The mean colony count was lowest in Group 1 (0.62 ± 0.23 × 10^3^ CFU/mL), intermediate in Group 2 (1.15 ± 0.59 × 10^3^ CFU/mL), and highest in Group 3 (1.82 ± 1.08 × 10^3^ CFU/mL). All pairwise comparisons were statistically significant.

### Association of SIBO with detectable HCV RNA, SVR status, and hepatic encephalopathy

Among cirrhotic patients, those with detectable HCV RNA had a significantly higher prevalence of SIBO compared to those without detectable HCV RNA (*p* < 0.001). Additionally, the median bacterial colony count was significantly greater in patients with detectable HCV RNA (*p* < 0.001). These findings indicate that ongoing HCV replication is associated with both increased SIBO prevalence and higher duodenal bacterial load (Table 5). All 24 patients with undetectable HCV RNA had achieved SVR after direct-acting antiviral agents (DAAs) therapy, whereas only 3 of 66 patients with detectable HCV RNA received DAAs but did not achieve SVR. Patients with SVR had a lower SIBO prevalence (20.8%) than DAA non-responders (66.7%), though this difference was not statistically significant (*p* = 0.16), likely due to limited sample size.

**Table 5:** Relation between detectable HCV RNA with SIBO & colony count in patients with cirrhosis

	Detectable HCV PCR		Test of sig	*p*
	No (n = 24)	Yes (n = 66)		
*SIBO*				
No	19 (97.2%)	22 (33.3%)	χ^2^ = 14.907	< 0.001
Yes	5 (20.8%)	44 (66.7%)		
*Colony count (*× *10*^*3*^* CFU/mL)*				
Min.–Max	0.4–3	0.3–4	U = 392.0	< 0.001
Mean ± SD	0.98 ± 0.61	1.74 ± 0.9		
Median (IQR)	0.8 (0.65–0.9)	1.9 (0.9–2.3)		

χ^2^: Chi-square test, IQR: Interquartile range, SD: Standard deviation, U: Mann–Whitney test

On the other hand, as shown in Table 6, no significant associations were found between the history of hepatic encephalopathy and either SIBO prevalence (*p* = 0.1) or bacterial colony count (*p* = 0.167).

**Table 6:** Relation between history of hepatic encephalopathy with SIBO & Colony count

	History of hepatic encephalopathy		Test of sig	*p*
	No (n = 60)	Yes (n = 30)		
*SIBO*				
No	31 (51.7%)	10 (33.3%)	χ^2^ = 2.71	0.1
Yes	29 (48.3%)	20 (66.7%)		
*Colony count (*× *10*^*3*^* CFU/mL.)*				
Min.–Max	0.3–4	0.4–4	U = 739	0.167
Mean ± SD	1.43 ± 0.86	1.74 ± 0.94		
Median (IQR)	1 (0.8–2.1)	1.9 (0.7–2.4)		

χ^2^: Chi-square test, IQR: Interquartile range, SD: Standard deviation, U: Mann–Whitney test

### Predictors of small intestinal bacterial overgrowth among cirrhotic patients

In univariate analysis, patients with SIBO were significantly older (mean age 56.8 vs. 50.6 years, *p* = 0.014) and had lower platelet counts (*p* = 0.002), higher bilirubin (*p* = 0.034), lower albumin (*p* = 0.004), higher Child–Pugh scores (*p* = 0.011), higher FIB-4 scores (*p* < 0.001), and higher MELD scores (*p* = 0.044) compared to those without SIBO. The presence of clinically evident portal hypertension was also significantly associated with SIBO (69.4% vs. 48.8%, *p* = 0.047). No significant differences were found in terms of gender, BMI, or liver stiffness measurements between the groups (Table 7).

**Table 7:** Baseline characteristics and liver disease severity stratified by SIBO status in patients with HCV-related cirrhosis

Variable	SIBO			Test of sig	*p*
	No (n = 41)	Yes (n = 49)			
*Gender*					
Male	23 (56.1%)	29 (59.2%)		χ^2^ = 0.087	0.768
Female	18 (43.9%)	20 (40.8%)			
*Age*					
Mean ± SD	50.59 ± 11.45	56.78 ± 11.84		t = 2.507	0.014
Median (Min.–Max.)	53.0(29.0–75.0)	59.0(22.0–76.0)			
BMI*					
Mean ± SD	24.99 ± 3.7	26.82 ± 5.16		t = 1.942	0.055
Median (Min.–Max.)	25.54 (17.9–34.64)	27.3 (18.9–36.2)			
Platelets (× 10^9^/L)					
Mean ± SD	147.78 ± 37.71	123.59 ± 34.69		t = 3.166	0.002
Median (Min.–Max.)	145.0 (84.0–221.0)	126.0 (50.0–198.0)			
*Total Bilirubin (mg/dL)*					
Mean ± SD	1.73 ± 1.10		2.01 ± 0.95	U = 743.50	0.034
Median (Min.–Max.)	1.20 (0.60–5.80)		2.0 (0.60–4.80)		
*Direct bilirubin (mg/dL)*					
Mean ± SD	1.01 ± 0.74		1.31 ± 0.86	U = 777.50	0.066
Median (Min.–Max.)	0.76 (0.10–3.50)		1.20 (0.16–4.70)		
*Albumin (gm/dL)*					
Mean ± SD	3.67 ± 0.67		3.26 ± 0.63	t = 2.988	0.004
Median (Min.–Max.)	3.60 (2.50–5.10)		3.10 (2.10–5.30)		
*AST (U/L)*					
Mean ± SD	45.05 ± 28.09		56.84 ± 29.80	U = 749.0	0.038
Median (Min.–Max.)	31.0 (15.0–123.0)		51.0 (16.0–146.0)		
*ALT (U/L)*					
Mean ± SD	37.12 ± 20.54		44.16 ± 31.42	U = 937.0	0.584
Median (Min.–Max.)	30.0 (11.0–87.0)		34.0 (11.0–170.0)		
*INR*					
Mean ± SD	1.32 ± 0.37		1.47 ± 0.39	U = 780.0	0.068
Median (Min.–Max.)	1.20 (0.80–2.17)		1.48 (0.80–2.50)		
*Creatinine (mg/dL)*					
Mean ± SD	1.15 ± 0.49		1.27 ± 0.56	U = 895.0	0.373
Median (Min.–Max.)	1.10 (0.60–3.20)		1.10 (0.60–2.90)		
*Child- Pugh score*					
Mean ± SD	6.66 ± 1.87	7.69 ± 1.78		U = 698	0.011
Median (Min.–Max.)	6.0 (5.0–10.0)	8.0 (5.0–11.0)			
*Child- Pugh*					
A	22 (53.7%)	12 (24.5%)		χ^2^ = 8.082	0.018
B	15 (36.6%)	29 (59.2%)			
C	4 (9.8%)	8 (16.3%)			
*FIB4*					
Mean ± SD	3.27 ± 1.93	4.92 ± 2.65		U = 569	< 0.001
Median (Min.–Max.)	2.50 (0.90–9.70)	4.40 (1.70–11.70)			
*MELD*					
Mean ± SD	12.56 ± 5.49	15.04 ± 5.47		U = 756.5	0.044
Median (Min.–Max.)	11.0 (6.0–26.0)	15.0 (6.0–27.0)			
*Liver stiffness (Kpa)*					
Mean ± SD	20.21 ± 4.95	21.45 ± 3.84		U = 688.0	0.114
Median (Min.–Max.)	19.0 (14.0–33.0)	22.0 (15.0–33.0)			
*Portal hypertension*					
No	21 (51.2%)	15 (30.6%)		χ^2^ = 3.95	0.047
Yes	20 (48.8%)	34 (69.4%)			

SD: Standard deviation, t: Student t-test, U: Mann–Whitney test, χ^2^: Chi-square test

BMI: Body mass index, *: BMI was calculated using estimated dry weight in ascitic patients, FIB-4: Fibrosis-4 index, MELD: Model for End-stage Liver Disease

In multivariate logistic regression analysis using a backward stepwise method, four independent predictors of SIBO emerged: age, FIB-4 score, MELD score, and the presence of portal hypertension. Age was significantly associated with higher odds of SIBO (OR = 1.091, 95% CI: 1.032–1.153, *p* = 0.002). Higher FIB-4 scores were significantly associated with increased SIBO risk (OR = 1.609, 95% CI 1.222–2.118, *p* = 0.001), as were higher MELD scores (OR = 1.145, 95% CI: 1.042–1.259, *p* = 0.005). Importantly, the presence of portal hypertension independently predicted the occurrence of SIBO (OR = 2.89, 95% CI 1.023–8.284, *p* = 0.048), indicating that patients with portal hypertension were nearly three times more likely to have SIBO compared to those without (Table 8). Child–Pugh score and platelet count were excluded from the final model due to a lack of statistical significance. The final model demonstrated good discriminative performance, with a classification accuracy of 77.8%.

**Table 8:** Multivariate logistic regression analysis of independent predictors of SIBO in cirrhotic patients

Variable	B	SE	Wald	*p*-value	OR (Exp(B))	95% CI for OR (approx.)
Age (years)	0.087	0.029	9.154	0.002	1.091	1.031–1.155
FIB-4	0.476	0.147	10.531	0.001	1.609	1.207–2.145
MELD Score	0.136	0.049	7.781	0.005	1.145	1.041–1.26
Portal Hypertension	1.061	0.542	3.828	0.048	2.890	1.023–8.284
Constant	− 9.000	2.278	15.602	< 0.001	–	–

B: regression coefficient, SE: standard error, Wald: Wald chi-square test statistic, OR: odds ratio, CI: confidence interval, FIB-4: Fibrosis-4 index, MELD: Model for End-stage Liver Disease

## Discussion

This study provides robust evidence for the high prevalence of SIBO among patients with HCV-related cirrhosis, particularly those with portal hypertension. Using duodenal aspirate cultures, the gold standard for SIBO diagnosis, we found SIBO in 63% of cirrhotic patients with portal hypertension, significantly higher than in cirrhotic patients without portal hypertension (41.7%) and non-liver disease controls (6.7%).

These findings are consistent with previous research showing high SIBO prevalence in patients with CLD and cirrhosis [23]. The overall prevalence in our cirrhotic cohort (54.4%) aligns with recent meta-analyses reporting pooled SIBO rates of 42.9% in cirrhosis [2]. This meta-analysis also estimated an eightfold increase in SIBO prevalence among CLD patients compared to healthy controls and showed significantly higher rates in decompensated compared to compensated cirrhosis (OR = 2.6; 95% CI 1.5–4.5), reinforcing the link between disease severity and gut dysbiosis [2].

The higher prevalence of SIBO observed in the portal hypertension group in our study supports the findings of Gulyaeva et al., who demonstrated that portal hypertension, independent of cirrhosis, plays a key role in driving significant gut microbial alterations [4].

The interplay between SIBO and portal hypertension is multifactorial. Portal hypertension promotes intestinal congestion, impaired motility, and mucosal edema, all of which disrupt the gut barrier and facilitate bacterial overgrowth and translocation [24, 25]. These changes exacerbate systemic inflammation and may further worsen portal hypertension, creating a vicious cycle [26, 27]. Our findings, showing that SIBO prevalence and bacterial colony counts were highest in patients with portal hypertension, support this pathophysiological model.

Multivariate logistic regression analysis identified age, FIB-4 score, MELD score, and the presence of portal hypertension as independent predictors of SIBO in cirrhotic patients. Each one-year increase in age was associated with 9.1% higher odds of SIBO (OR = 1.091, 95% CI 1.032–1.153, *p* = 0.002). This suggests that older age is a significant risk factor for SIBO in this population, possibly due to age-related changes in gut motility and immune function. The FIB-4 score (OR = 1.609, 95% CI 1.207–2.145, *p* = 0.001) and MELD score (OR = 1.145, 95% CI 1.041–1.26, *p* = 0.005) are established indicators of liver fibrosis and disease severity, respectively. Their independent association with SIBO further strengthens the link between advanced liver disease and bacterial overgrowth. This is in line with previous studies demonstrating that positive non-invasive fibrosis markers and elevated liver disease severity scores are significantly associated with an increased risk of SIBO [28–30].

Notably, portal hypertension was associated with nearly threefold increased odds of SIBO (OR = 2.89, 95% CI 1.02–8.28, *p* = 0.048), even after adjusting for liver disease severity and other confounders. This aligns with prior research suggesting a strong link between portal hypertension and intestinal dysbiosis [4, 31]. The borderline statistical significance of this association may reflect the sample size and inherent variability in clinical presentations, but the effect size underscores its clinical relevance. The identification of these predictors can aid in the early identification of cirrhotic patients at high risk for SIBO, allowing for timely interventions.

The analysis of bacterial species in duodenal aspirates revealed distinct microbial profiles across the study groups. Bacteroides and co-isolation of Escherichia coli and Lactobacillus were exclusively observed in the control group. Lactobacilli were significantly more prevalent in Group 1 (*p* < 0.001), suggesting their role as beneficial commensals in a healthy gut environment. Conversely, Enterococcus faecalis was significantly more common in cirrhotic patients, particularly in Groups 2 and 3 (*p* = 0.009), while Streptococci predominated in Group 2 (*p* = 0.013). The presence of other organisms such as Klebsiella, Pseudomonas, Serratia, and Candida exclusively in Group 3 (cirrhosis with portal hypertension) further indicates a shift towards a more pathogenic and diverse microbial community in advanced liver disease. This dysbiosis, characterized by an increase in potentially harmful bacteria and a reduction in beneficial ones, aligns with existing literature on gut microbiota alterations in cirrhosis [32]. The increased presence of Enterococcus and Streptococcus species, as well as Gram-negative bacteria like Klebsiella and Pseudomonas, is particularly concerning given their association with bacterial translocation and systemic inflammation, which can exacerbate portal hypertension and its complications [33].

This shift toward a more pathogenic microbiome in portal hypertension mirrors observations by Bajaj et al. in hepatic encephalopathy [34], suggesting a shared dysbiotic pathway exacerbated by portal hypertension. This dysbiotic profile is consistent with previous microbiome studies in CLD and may contribute to the pathogenesis of liver-related complications [35–37].

Our study found significant differences in bacterial colony counts among the groups (*p* < 0.001), with the lowest mean count in Group 1 (0.62 ± 0.23 × 10^3^ CFU/mL), intermediate in Group 2 (1.15 ± 0.59 × 10^3^ CFU/mL), and highest in Group 3 (1.82 ± 1.08 × 10^3^ CFU/mL). This clear gradient in bacterial load, directly correlating with the presence of portal hypertension and severity of liver disease, underscores the pathological significance of SIBO in cirrhotic patients. Higher bacterial loads contribute to increased intestinal permeability and bacterial translocation, leading to systemic inflammation and endothelial dysfunction, which can further aggravate portal hypertension [32]. The use of duodenal aspirate cultures allowed for a precise quantification of bacterial load, providing a more accurate assessment compared to less specific diagnostic methods. These findings emphasize the importance of not only identifying the presence of SIBO but also quantifying the bacterial burden to better understand its impact on disease progression [19, 38, 39].

Our findings demonstrate that detectable HCV RNA is significantly associated with both the presence of SIBO and increased duodenal bacterial colony counts in cirrhotic patients. This suggests that ongoing viral replication may contribute to gut dysbiosis, potentially through mechanisms such as immune activation, mucosal barrier dysfunction, or altered bile acid metabolism, all of which can favor bacterial overgrowth [40, 41]. These results are in line with previous studies indicating that active HCV infection exacerbates intestinal permeability and microbial translocation, thereby increasing the risk of SIBO and its complications [42–45].

Eradication of HCV has been reported to improve portal pressure, systemic inflammation, and gut barrier function, factors that may reduce susceptibility to SIBO [46–48]. Our data suggest a trend towards lower SIBO prevalence among patients with SVR, highlighting a potential modifying effect of viral clearance on intestinal bacterial overgrowth. Larger studies are warranted to confirm these findings and to evaluate SIBO as a modifiable target in patients successfully treated for HCV.

Interestingly, a history of hepatic encephalopathy was not associated with SIBO in our cohort. Although previous studies have reported a positive association between SIBO and hepatic encephalopathy [3, 49–51], our negative finding may be attributed to the modest sample size or the multifactorial etiology of encephalopathy in patients with cirrhosis.

These results have important clinical implications. First, they underscore the potential value of routine SIBO screening in cirrhotic patients, particularly those with portal hypertension or elevated non-invasive fibrosis scores. Early detection and treatment may help reduce complications such as hepatic encephalopathy and spontaneous bacterial peritonitis, which are driven by bacterial translocation and gut-derived inflammation [52]. Second, our findings support the use of non-invasive markers (FIB-4, MELD) for risk stratification and guiding targeted interventions. Third, from a translational perspective, the identification of SIBO as a significant factor in HCV-related cirrhosis, especially in the presence of portal hypertension, highlights the potential for microbiome-targeted therapeutic strategies. Non-absorbable antibiotics (e.g., rifaximin), specific probiotics, and dietary modulation have shown promise in improving gut barrier function, restoring microbial balance, and reducing bacterial translocation in patients with cirrhosis [53–55]. While our study did not assess the efficacy of such interventions, our findings provide a strong rationale for integrating SIBO evaluation and management into comprehensive care pathways for this patient population.

A major strength of this study is the use of duodenal aspirate culture, a more accurate diagnostic method than breath tests. The inclusion of well-defined patient groups and comprehensive clinical and microbiological assessments enhances the validity of our findings.

### Limitations

Several limitations should be acknowledged. The most important is the cross-sectional design, which precludes establishing causality and assessing long-term clinical outcomes. We were unable to determine whether SIBO predicts important clinical events such as hepatic decompensation, infections, hospitalizations, or transplant-free survival. The relatively small sample size, although adequate to detect significant associations, may limit generalizability. Our study also did not evaluate gastrointestinal symptoms associated with SIBO, limiting symptom–microbiology correlations. Additionally, dietary data were not collected, representing a missed opportunity to assess an important modulator of gut microbiota. The borderline significance of portal hypertension as a predictor of SIBO requires confirmation in larger, multicenter cohorts. Exclusion of patients with diabetes, recent antibiotic use, active alcohol intake, or other confounding factors, while methodologically necessary, may limit applicability to broader cirrhotic populations. Finally, as the study was restricted to HCV-related cirrhosis, findings may not apply to other etiologies.

### Future directions

Future research should focus on conducting large, multicenter longitudinal studies to clarify the temporal relationship between SIBO and liver disease progression. Interventional trials are needed to determine whether targeted SIBO screening and treatment can improve outcomes in advanced liver disease. Given that duodenal aspiration is invasive and often impractical in routine settings, efforts should be directed toward developing and validating less invasive yet accurate diagnostic alternatives. Further studies should also explore the contribution of specific bacterial species to the pathophysiology of portal hypertension and expand investigations to other cirrhosis etiologies. Incorporating validated symptom assessment tools will help strengthen correlations between microbiological findings and clinical presentation.

## Conclusion

In conclusion, our study demonstrates a high prevalence of SIBO in HCV-related cirrhosis, especially among patients with portal hypertension. Age, FIB-4 score, MELD score, and portal hypertension were independent predictors of SIBO. The observed microbial shifts and increased bacterial load highlight the role of gut dysbiosis in liver disease progression. These findings underscore the need to consider SIBO in the clinical management of cirrhotic patients with portal hypertension and support the use of non-invasive liver disease markers for SIBO risk stratification. Given the cross-sectional nature and other limitations noted, further longitudinal studies are needed to elucidate causality and clinical implications, including the potential benefits of therapeutic interventions targeting SIBO in this population.

## Data Availability

The datasets used in the current study are available from the corresponding author upon reasonable request.

## References

[1] Liu Y, Chen Z, Li C, Sun T, Luo X, Jiang B, et al. Associations between changes in the gut microbiota and liver cirrhosis: a systematic review and meta-analysis. BMC Gastroenterol. 2025;25:16. 10.1186/s12876-025-03589-5. 39806278 10.1186/s12876-025-03589-5 PMC11727502

[2] Shah A, Spannenburg L, Thite P, Morrison M, Fairlie T, Koloski N, et al. Small intestinal bacterial overgrowth in chronic liver disease: an updated systematic review and meta-analysis of case-control studies. EClinicalMedicine. 2025;80:103024. 10.1016/j.eclinm.2024.103024. 39844931 10.1016/j.eclinm.2024.103024 PMC11751576

[3] Sakamaki A, Yokoyama K, Yamazaki H, Wakabayashi T, Kojima Y, Tominaga K, et al. Small intestinal bacterial overgrowth is a predictor of overt hepatic encephalopathy in patients with liver cirrhosis. J Clin Med. 2025. 10.3390/jcm14051491. 40094949 10.3390/jcm14051491 PMC11901010

[4] Gulyaeva K, Nadinskaia M, Maslennikov R, Aleshina Y, Goptar I, Lukashev A, et al. Gut microbiota analysis in cirrhosis and non-cirrhotic portal hypertension suggests that portal hypertension can be main factor of cirrhosis-specific dysbiosis. Sci Rep. 2025;15:8394. 10.1038/s41598-025-92618-0. 40069378 10.1038/s41598-025-92618-0 PMC11897210

[5] Alexiou O, Despotis G, Kalambokis G, Tsiakas I, Christaki M, Tsiouris S, et al. Impact of small intestinal bacterial overgrowth on systemic inflammation, circulatory and renal function, and liver fibrosis in patients with cirrhosis and ascites. Ann Gastroenterol. 2024;37:348–55. 10.20524/aog.2024.0881. 38779647 10.20524/aog.2024.0881 PMC11107405

[6] Shah ED. Breath test or duodenal aspirate for small intestinal bacterial overgrowth: still no breath of fresh air. Dig Dis Sci. 2021;66:1770–1. 10.1007/s10620-020-06556-0. 32816209 10.1007/s10620-020-06556-0

[7] Atsukawa M, Tsubota A, Kondo C, Uchida-Kobayashi S, Takaguchi K, Tsutsui A, et al. A novel noninvasive formula for predicting cirrhosis in patients with chronic hepatitis C. PLoS ONE. 2021;16:e0257166. 10.1371/journal.pone.0257166. 34506563 10.1371/journal.pone.0257166 PMC8432856

[8] Castera L, Vergniol J, Foucher J, Le Bail B, Chanteloup E, Haaser M, et al. Prospective comparison of transient elastography, Fibrotest, APRI, and liver biopsy for the assessment of fibrosis in chronic hepatitis C. Gastroenterology. 2005;128:343–50. 10.1053/j.gastro.2004.11.018. 15685546 10.1053/j.gastro.2004.11.018

[9] Thabut D, Bureau C, Layese R, Bourcier V, Hammouche M, Cagnot C, et al. Validation of baveno VI criteria for screening and surveillance of esophageal varices in patients with compensated cirrhosis and a sustained response to antiviral therapy. Gastroenterology. 2019;156(997–1009):e5. 10.1053/j.gastro.2018.11.053. 10.1053/j.gastro.2018.11.053 30768988

[10] European Association for the Study of the Liver. EASL recommendations on treatment of hepatitis C: Final update of the series(☆). J Hepatol. 2020;73:1170–218. 10.1016/j.jhep.2020.08.018. (**Electronic address eee, Clinical Practice Guidelines Panel C, representative EGB, Panel m.**). 32956768 10.1016/j.jhep.2020.08.018

[11] European Association for the Study of the L. EASL Clinical Practice Guidelines on nutrition in chronic liver disease. J Hepatol. 2019;70:172–93. 10.1016/j.jhep.2018.06.024. 10.1016/j.jhep.2018.06.024 PMC6657019 30144956

[12] Tandon P, Low G, Mourtzakis M, Zenith L, Myers RP, Abraldes JG, et al. A Model to Identify Sarcopenia in Patients With Cirrhosis. Clin Gastroenterol Hepatol. 2016;14(1473–80):e3. 10.1016/j.cgh.2016.04.040. 27189915 10.1016/j.cgh.2016.04.040

[13] European Association for the Study of the L. EASL Clinical Practice Guidelines for the management of patients with decompensated cirrhosis. J Hepatol. 2018;69:406–60. 10.1016/j.jhep.2018.03.024. 10.1016/j.jhep.2018.03.024 29653741

[14] de Franchis R, Bosch J, Garcia-Tsao G, Reiberger T, Ripoll C, Baveno VIIF. Baveno VII - Renewing consensus in portal hypertension. J Hepatol. 2022;76:959–74. 10.1016/j.jhep.2021.12.022. 35120736 10.1016/j.jhep.2021.12.022 PMC11090185

[15] Pugh RN, Murray-Lyon IM, Dawson JL, Pietroni MC, Williams R. Transection of the oesophagus for bleeding oesophageal varices. Br J Surg. 1973;60:646–9. 10.1002/bjs.1800600817. 4541913 10.1002/bjs.1800600817

[16] Kamath PS, Wiesner RH, Malinchoc M, Kremers W, Therneau TM, Kosberg CL, et al. A model to predict survival in patients with end-stage liver disease. Hepatology. 2001;33:464–70. 10.1053/jhep.2001.22172. 11172350 10.1053/jhep.2001.22172

[17] Sterling RK, Lissen E, Clumeck N, Sola R, Correa MC, Montaner J, et al. Development of a simple noninvasive index to predict significant fibrosis in patients with HIV/HCV coinfection. Hepatology. 2006;43:1317–25. 10.1002/hep.21178. 16729309 10.1002/hep.21178

[18] Paquet KJ. Prophylactic endoscopic sclerosing treatment of the esophageal wall in varices – a prospective controlled randomized trial. Endoscopy. 1982;14:4–5. 10.1055/s-2007-1021560. 7035153 10.1055/s-2007-1021560

[19] Franco DL, Disbrow MB, Kahn A, Koepke LM, Harris LA, Harrison ME, et al. Duodenal aspirates for small intestine bacterial overgrowth: yield, PPIs, and Outcomes after treatment at a tertiary academic medical center. Gastroenterol Res Pract. 2015;2015:971582. 10.1155/2015/971582. 25694782 10.1155/2015/971582 PMC4324922

[20] Silva BCD, Ramos GP, Barros LL, Ramos AFP, Domingues G, Chinzon D, et al. Diagnosis and treatment of small intestinal bacterial overgrowth: an official position paper from the Brazilian Federation of Gastroenterology. Arq Gastroenterol. 2025;62:e24107. 10.1590/S0004-2803.24612024-107. 39968993 10.1590/S0004-2803.24612024-107 PMC12043196

[21] Rezaie A, Buresi M, Lembo A, Lin H, McCallum R, Rao S, et al. Hydrogen and methane-based breath testing in gastrointestinal disorders: the North American consensus. Am J Gastroenterol. 2017;112:775–84. 10.1038/ajg.2017.46. 28323273 10.1038/ajg.2017.46 PMC5418558

[22] Pimentel M, Saad RJ, Long MD, Rao SSC. ACG clinical guideline: small intestinal bacterial overgrowth. Am J Gastroenterol. 2020;115:165–78. 10.14309/ajg.0000000000000501. 32023228 10.14309/ajg.0000000000000501

[23] Maslennikov R, Pavlov C, Ivashkin V. Small intestinal bacterial overgrowth in cirrhosis: systematic review and meta-analysis. Hepatol Int. 2018;12:567–76. 10.1007/s12072-018-9898-2. 30284684 10.1007/s12072-018-9898-2

[24] Baffy G. Potential mechanisms linking gut microbiota and portal hypertension. Liver Int. 2019;39:598–609. 10.1111/liv.13986. 30312513 10.1111/liv.13986

[25] Gunnarsdottir SA, Sadik R, Shev S, Simren M, Sjovall H, Stotzer PO, et al. Small intestinal motility disturbances and bacterial overgrowth in patients with liver cirrhosis and portal hypertension. Am J Gastroenterol. 2003;98:1362–70. 10.1111/j.1572-0241.2003.07475.x. 12818282 10.1111/j.1572-0241.2003.07475.x

[26] Ghosh G, Jesudian AB. Small intestinal bacterial overgrowth in patients with cirrhosis. J Clin Exp Hepatol. 2019;9:257–67. 10.1016/j.jceh.2018.08.006. 31024208 10.1016/j.jceh.2018.08.006 PMC6477138

[27] Fukui H, Wiest R. Changes of intestinal functions in liver cirrhosis. Inflamm Intest Dis. 2016;1:24–40. 10.1159/000444436. 29922655 10.1159/000444436 PMC5988129

[28] Maslennikov R, Ivashkin V, Efremova I, Poluektova E, Kudryavtseva A, Krasnov G. Gut dysbiosis and small intestinal bacterial overgrowth as independent forms of gut microbiota disorders in cirrhosis. World J Gastroenterol. 2022;28(10):1067–77. 10.3748/wjg.v28.i10.1067. 35431497 10.3748/wjg.v28.i10.1067 PMC8968519

[29] Mikolasevic I, Delija B, Mijic A, Stevanovic T, Skenderevic N, Sosa I, et al. Small intestinal bacterial overgrowth and non-alcoholic fatty liver disease diagnosed by transient elastography and liver biopsy. Int J Clin Pract. 2021;75:e13947. 10.1111/ijcp.13947. 33406286 10.1111/ijcp.13947

[30] Pande C, Kumar A, Sarin SK. Small-intestinal bacterial overgrowth in cirrhosis is related to the severity of liver disease. Aliment Pharmacol Ther. 2009;29:1273–81. 10.1111/j.1365-2036.2009.03994.x. 19302262 10.1111/j.1365-2036.2009.03994.x

[31] Pun CK, Huang HC, Chang CC, Chuang CL, Hsu SJ, Hou MC, et al. Fructooligosaccharides reverses hepatic vascular dysfunction and dysbiosis in rats with liver cirrhosis and portal hypertension. Eur J Clin Invest. 2024;54:e14287. 10.1111/eci.14287. 39017981 10.1111/eci.14287

[32] Ponziani FR, Zocco MA, Cerrito L, Gasbarrini A, Pompili M. Bacterial translocation in patients with liver cirrhosis: physiology, clinical consequences, and practical implications. Expert Rev Gastroenterol Hepatol. 2018;12:641–56. 10.1080/17474124.2018.1481747. 29806487 10.1080/17474124.2018.1481747

[33] Bellot P, Frances R, Such J. Pathological bacterial translocation in cirrhosis: pathophysiology, diagnosis and clinical implications. Liver Int. 2013;33:31–9. 10.1111/liv.12021. 23121656 10.1111/liv.12021

[34] Bajaj JS, Hylemon PB, Ridlon JM, Heuman DM, Daita K, White MB, et al. Colonic mucosal microbiome differs from stool microbiome in cirrhosis and hepatic encephalopathy and is linked to cognition and inflammation. Am J Physiol Gastrointest Liver Physiol. 2012;303:G675–85. 10.1152/ajpgi.00152.2012. 22821944 10.1152/ajpgi.00152.2012 PMC3468538

[35] Chen Y, Yang F, Lu H, Wang B, Chen Y, Lei D, et al. Characterization of fecal microbial communities in patients with liver cirrhosis. Hepatology. 2011;54:562–72. 10.1002/hep.24423. 21574172 10.1002/hep.24423

[36] Oh TG, Kim SM, Caussy C, Fu T, Guo J, Bassirian S, et al. A universal gut-microbiome-derived signature predicts cirrhosis. Cell Metab. 2020;32(878–88):e6. 10.1016/j.cmet.2020.06.005. 10.1016/j.cmet.2020.06.005 PMC7822714 32610095

[37] Shao L, Ling Z, Chen D, Liu Y, Yang F, Li L. Disorganized gut microbiome contributed to liver cirrhosis progression: a meta-omics-based study. Front Microbiol. 2018;9:3166. 10.3389/fmicb.2018.03166. 30631318 10.3389/fmicb.2018.03166 PMC6315199

[38] Cangemi DJ, Lacy BE, Wise J. Diagnosing small intestinal bacterial overgrowth: a comparison of lactulose breath tests to small bowel aspirates. Dig Dis Sci. 2021;66:2042–50. 10.1007/s10620-020-06484-z. 32681227 10.1007/s10620-020-06484-z

[39] Erdogan A, Rao SS, Gulley D, Jacobs C, Lee YY, Badger C. Small intestinal bacterial overgrowth: duodenal aspiration vs glucose breath test. Neurogastroenterol Motil. 2015;27:481–9. 10.1111/nmo.12516. 25600077 10.1111/nmo.12516

[40] Inoue T, Funatsu Y, Ohnishi M, Isogawa M, Kawashima K, Tanaka M, et al. Bile acid dysmetabolism in the gut-microbiota-liver axis under hepatitis C virus infection. Liver Int. 2022;42:124–34. 10.1111/liv.15041. 34411400 10.1111/liv.15041

[41] El-Mowafy M, Elgaml A, El-Mesery M, Sultan S, Ahmed TAE, Gomaa AI, et al. Changes of gut-microbiota-liver axis in hepatitis C virus infection. Biol. 2021. 10.3390/biology10010055. 10.3390/biology10010055 PMC7828638 33451143

[42] Preveden T, Scarpellini E, Milic N, Luzza F, Abenavoli L. Gut microbiota changes and chronic hepatitis C virus infection. Expert Rev Gastroenterol Hepatol. 2017;11:813–9. 10.1080/17474124.2017.1343663. 28621554 10.1080/17474124.2017.1343663

[43] Chuaypen N, Jinato T, Avihingsanon A, Nookaew I, Tanaka Y, Tangkijvanich P. Long-term benefit of DAAs on gut dysbiosis and microbial translocation in HCV-infected patients with and without HIV coinfection. Sci Rep. 2023;13:14413. 10.1038/s41598-023-41664-7. 37660163 10.1038/s41598-023-41664-7 PMC10475021

[44] Marascio N, De Caro C, Quirino A, Mazzitelli M, Russo E, Torti C, et al. The role of the microbiota gut-liver axis during HCV chronic infection: a schematic overview. J Clin Med. 2022. 10.3390/jcm11195936. 36233804 10.3390/jcm11195936 PMC9572099

[45] Hsu YC, Chen CC, Lee WH, Chang CY, Lee FJ, Tseng CH, et al. Compositions of gut microbiota before and shortly after hepatitis C viral eradication by direct antiviral agents. Sci Rep. 2022;12:5481. 10.1038/s41598-022-09534-w. 35361930 10.1038/s41598-022-09534-w PMC8971444

[46] Perez-Matute P, Iniguez M, Villanueva-Millan MJ, Recio-Fernandez E, Vazquez AM, Sanchez SC, et al. Short-term effects of direct-acting antiviral agents on inflammation and gut microbiota in hepatitis C-infected patients. Eur J Intern Med. 2019;67:47–58. 10.1016/j.ejim.2019.06.005. 31221551 10.1016/j.ejim.2019.06.005

[47] Ponziani FR, Putignani L, Paroni Sterbini F, Petito V, Picca A, Del Chierico F, et al. Influence of hepatitis C virus eradication with direct-acting antivirals on the gut microbiota in patients with cirrhosis. Aliment Pharmacol Ther. 2018;48:1301–11. 10.1111/apt.15004. 30345704 10.1111/apt.15004

[48] Armandi A, Rosso C, Troshina G, Del Campo NPD, Marinoni C, Nicolosi A, et al. Changes in liver stiffness and markers of liver synthesis and portal hypertension following hepatitis C virus eradication in cirrhotic individuals. Biology (Basel). 2022. 10.3390/biology11081160. 36009789 10.3390/biology11081160 PMC9404889

[49] Feng X, Li X, Zhang X, Chen W, Tian Y, Yang Q, et al. Hepatic encephalopathy in cirrhotic patients and risk of small intestinal bacterial overgrowth: a systematic review and meta-analysis. Biomed Res Int. 2022;2022:2469513. 10.1155/2022/2469513. 36303585 10.1155/2022/2469513 PMC9596239

[50] Yokoyama K, Sakamaki A, Takahashi K, Naruse T, Sato C, Kawata Y, et al. Hydrogen-producing small intestinal bacterial overgrowth is associated with hepatic encephalopathy and liver function. PLoS ONE. 2022;17:e0264459. 10.1371/journal.pone.0264459. 35213654 10.1371/journal.pone.0264459 PMC8880851

[51] Zhang Y, Feng Y, Cao B, Tian Q. The effect of small intestinal bacterial overgrowth on minimal hepatic encephalopathy in patients with cirrhosis. Arch Med Sci. 2016;12:592–6. 10.5114/aoms.2015.55675. 27279853 10.5114/aoms.2015.55675 PMC4889679

[52] Hegazy RA. Unraveling liver cirrhosis: bridging pathophysiology to innovative therapeutics. J Gastroenterol Hepatol. 2025. 10.1111/jgh.70037. 40754005 10.1111/jgh.70037

[53] Stachowska E, Gudan A, Mankowska-Wierzbicka D, Liebe R, Krawczyk M. Dysbiosis and nutrition in steatotic liver disease: addressing the unrecognized small intestinal bacterial overgrowth (SIBO) challenge. Intern Emerg Med. 2024;19:1229–34. 10.1007/s11739-024-03533-7. 38499938 10.1007/s11739-024-03533-7

[54] Pisarello MJL, Marquez A, Chaia AP, Babot JD. Targeting gut health: probiotics as promising therapeutics in alcohol-related liver disease management. AIMS Microbiol. 2025;11:410–35. 10.3934/microbiol.2025019. 40600214 10.3934/microbiol.2025019 PMC12207258

[55] Yu JX, Wu J, Chen X, Zang SG, Li XB, Wu LP, et al. Gut microbiota in liver diseases: initiation, development and therapy. Front Med Lausanne. 2025;12:1615839. 10.3389/fmed.2025.1615839. 40534699 10.3389/fmed.2025.1615839 PMC12174154

